# Asymptomatic Common Bile Duct Stones Are Associated with Increased Risk of Post-Endoscopic Retrograde Cholangiopancreatography Pancreatitis

**DOI:** 10.31662/jmaj.2020-0123

**Published:** 2021-03-26

**Authors:** Makoto Kadokura, Yumi Takenaka, Hiroki Yoda, Tomoki Yasumura, Tetsuya Okuwaki, Keisuke Tanaka, Fumitake Amemiya

**Affiliations:** 1 Department of Gastroenterology, Kofu Municipal Hospital, Kofu, Japan

**Keywords:** asymptomatic, common bile duct stones, deep cannulation time, endoscopic papillary balloon dilation, post-ERCP pancreatitis

## Abstract

**Introduction:** Common bile duct stones (CBDS) are a common disease that can cause biliary complications, including cholangitis, obstructive jaundice, and biliary pancreatitis. Regardless of the presence or absence of symptoms, endoscopic removal of CBDS is generally recommended, but endoscopic retrograde cholangiopancreatography (ERCP) is a high-risk procedure with complications, such as post-ERCP pancreatitis (PEP). As few reports have addressed the risk of PEP by focusing on asymptomatic CBDS, the purpose of this study is to examine the incidence of PEP for asymptomatic CBDS.

**Methods:** This retrospective study included data from 302 patients with naive papilla who underwent therapeutic ERCP for CBDS between January 2012 and December 2019 at our hospital. Univariate and multivariate logistic regression models were used to investigate independent risk factors for PEP.

**Results:** Of the 302 patients, 32 were asymptomatic, and the remaining 270 were symptomatic. Five asymptomatic patients (15.6%) suffered from mild PEP, whereas 10 (3.7%) symptomatic patients suffered from PEP (9 were mild, and 1 was severe). Univariate analysis identified deep cannulation time more than 10 min, endoscopic papillary balloon dilation (EPBD), and asymptomatic CBDS as risk factors for PEP, whereas multivariate analysis revealed deep cannulation time more than 10 min (odds ratio (OR), 6.67; p < 0.001), EPBD (HR, 5.70; p < 0.001), and asymptomatic CBDS (HR, 5.49; p < 0.001) as independent risk factors for PEP.

**Conclusions:** A wait-and-see approach may be an option for the management of asymptomatic CBDS. EPBD may be avoided, especially in case of asymptomatic or if difficult for bile duct cannulation.

## Introduction

Endoscopic retrograde cholangiopancreatography (ERCP) is a useful procedure for the diagnosis and treatment of biliopancreatic diseases and is the initial treatment option for common bile duct stones (CBDS). Owing to advances in and the availability of abdominal imaging modalities, asymptomatic bile duct stones are being increasingly identiﬁed in clinical practice ^(1)^. Although the natural history of asymptomatic CBDS is unclear, it carries a risk of concurrent cholangitis and pancreatitis. Therefore, treatment is generally recommended in the guidelines of various countries ^(2), (3), (4)^, including Japan ^(5)^.

However, ERCP is a high-risk procedure that is associated with complications such as cholangitis, bleeding, perforation, and pancreatitis. Of these, post-ERCP pancreatitis (PEP) is the most common and serious complication as it can be fatal ^(6)^. Although endoscopic stone removal requires papillary treatment, including endoscopic sphincterotomy (EST), endoscopic papillary balloon dilation (EPBD), or endoscopic papillary large balloon dilation (EPLBD), these papillary treatments are also risk factors for the development of PEP ^(7), (8)^.

As ERCP is a high-risk procedure, indications for ERCP in CBDS, especially in asymptomatic patients, should be determined only after careful consideration of the accompanying risks and benefits, but few reports have evaluated the risk of PEP for asymptomatic CBDS ^(9), (10), (11), (12), (13)^. Therefore, the purpose of this study is to examine the incidence and severity of PEP in asymptomatic CBDS patients with a naive papilla and identify potential risk factors.

## Materials and Methods

### Study population, design, and data collection

Using medical records at our hospital for the period between January 2012 and December 2019, we identiﬁed patients who had CBDS diseases with native papilla and normal gastrointestinal tract or Billroth I gastrectomy and who underwent ERCP at our hospital. We excluded those who had biliary pancreatitis and undetected CBDS during ERCP. Thus, 302 patients (32 patients with asymptomatic CBDS and 270 patients with symptomatic CBDS) were included in the study. The institutional review board of Kofu Municipal Hospital approved this study (approval code 31-28). Informed consent was obtained in the form of opt-out on the website.

### Diagnostic procedure

One or more of the following imaging modalities were used to diagnose CBDS in all patients, namely, computed tomography (CT), magnetic resonance cholangiopancreatography (MRCP), ultrasonography (US), and cholangiography during laparoscopic cholecystectomy. If CBDS was not detected by imaging, it was diagnosed based on clinical findings, such as abdominal pain, elevated liver function tests, and/or dilated CBD. Diagnosis and grading of acute cholangitis were based on Tokyo guidelines 2018 ^(14)^.

### Endoscopist and therapeutic procedure

Of a total of 14 different endoscopists, 2 were experts, and 12 were trainees. Endoscopists were considered experts if they could perform procedures equivalent to Grade 3 of the grading scale for the difficulty of ERCP, based on the ERCP core curriculum ^(15)^, without assistance. Endoscopists were considered trainees if they had performed fewer than 200 ERCP procedures or could only perform procedures equivalent to Grade 1 with or without assistance. When performing the procedure, trainees were assisted by an expert.

Since April 2018, premedication of NSAIDs (diclofenac sodium 25 mg) has been routinely used to prevent PEP, except for contraindicated cases. Midazolam and pethidine hydrochloride were used for sedation, and scopolamine butyl bromide or glucagon was used for duodenal relaxation. We used side-viewing duodenoscopy (JF-260V; Olympus Medical Systems, Tokyo, Japan) in all patients. The primary biliary cannulation technique was the contrast injection method. If a trainee did not succeed deep cannulation within 5 min, change to an expert. A basket and/or a balloon catheter and/or a mechanical lithotriptor were used to remove CBDS. The type of papillary treatment (EPBD/EST/EPLBD) and choice of device were at the discretion of the endoscopist and depended on the size and number of stones and on the general condition of the patient. EPLBD was performed after minimal EST.

A biliary stent was inserted in patients with cholangitis who want to finish treatment quickly or in patients who had a large stone or had multiple stones and were considered not suitable for either repeated ERCP or surgical intervention (e.g., elderly or frail patient with multiple comorbidities).

### Study definitions

Asymptomatic CBDS was defined as the absence of symptoms and blood data associated with CBDS (total bilirubin, direct bilirubin, aspartate aminotransferase/alanine aminotransferase, γ-glutamyltransferase, alkaline phosphatase, white blood count, and C-reactive protein) at the time of ERCP. Symptomatic CBDS was defined as cholangitis, obstructive jaundice, or elevated liver function test values.

We deﬁned difﬁcult cannulation as procedures that required >10 min for deep cannulation because a cannulation attempt requiring >10 min has previously been reported as a deﬁnite risk factor for PEP ^(7)^.

Complications (including PEP) and its severity were deﬁned based on the criteria established by the American Society of Gastrointestinal Endoscopy ^(16)^.

### Statistical analysis

Associations between PEP and risk factors were assessed using chi-square or Fisher’s exact tests for categorical variables and using the Mann–Whitney U test for continuous variables. Risk factors with P values less than 0.1 in univariate analysis were used in logistic regression for multivariate analysis. A similar univariate analysis was performed to examine the association between symptom status (asymptomatic/symptomatic) and risk factors. All p values of < 0.05 obtained by a two-tailed test were considered significant. All statistical analyses were performed on EZR software (Saitama Medical Center, Jichi Medical University, Saitama, Japan), a graphical user interface for R (The R Foundation for Statistical Computing, Vienna, Austria). More precisely, it is a modified version of the R commander designed to add the statistical functions frequently used in biostatistics ^(17)^.

## Results

### Patient characteristics

**Figure 1.**
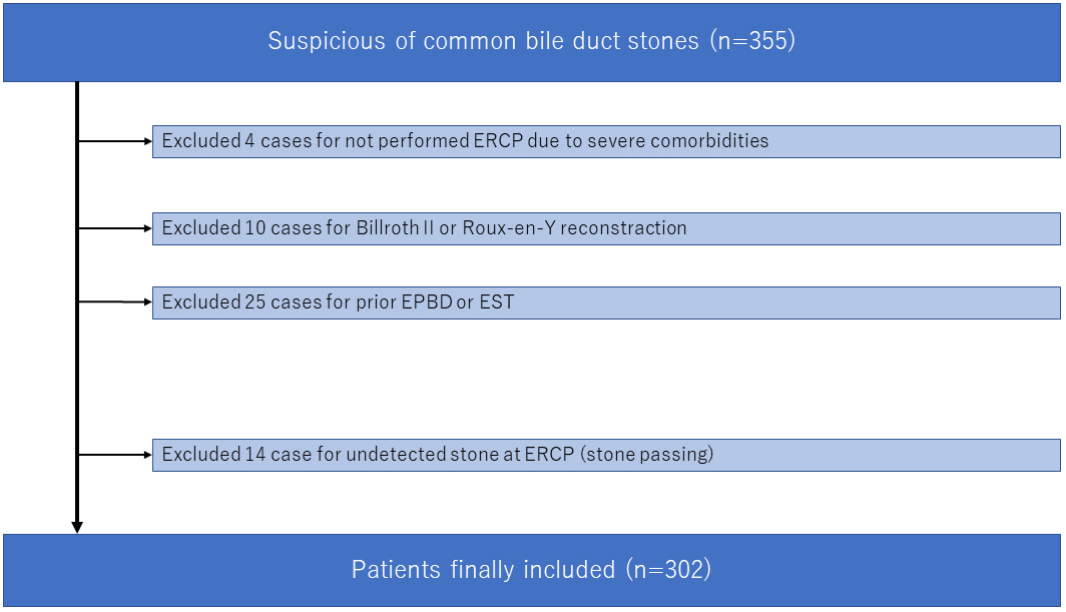
Flowchart of patient selection. ERCP, endoscopic retrograde cholangiopancreatography; EPBD, endoscopic papillary balloon dilation; EST, endoscopic sphincterotomy.

We identified 355 patients diagnosed with CBDS. After excluding 53 patients (Figure 1), 302 CBDS patients were included in the study, of whom 32 were asymptomatic and the remaining 270 were symptomatic.

The details of both symptomatic and asymptomatic patients, including demographics and other characteristics, are listed in Table 1. There were no significant differences between asymptomatic and symptomatic patients, except for the proportion of patients aged under 55 and serum bilirubin level.

**Table 1.** Patient Characteristics.

Category	Asymptomatic (n = 32)	Symptomatic (n = 270)	*p* value
Female sex (%)	11 (34.4)	121 (44.8)	0.35^*^
Age < 55 years (%)	0	36 (13.3)	***0.02***
Maximum Stone Diameter ≥ 10 mm (%)	7 (21.9)	65 (24.1)	1
Number of Stones ≥ 2 (%)	15 (46.9)	135 (50)	0.85
Diameter of Common Bile Duct < 10 mm (%)	20 (62.5)	184 (68.1)	0.55
Serum Bilirubin < 1.5 mg/dL (%)	28 (87.5)	132 (48.9)	***< 0.001***
No Pre-Procedural Diclofenac (%)	28 (87.5)	237 (87.8)	1
Precut Sphincterotomy (%)	2 (6.3)	10 (3.7)	0.37
Deep Cannulation Time ≥ 10 min (%)	10 (31.3)	105 (38.9)	0.447
Papillary Treatment EPBD (%)	12 (37.5)	86 (31.9)	0.55
Contrast Injection into Pancreatic Duct (%)	17 (53.1)	150 (55.6)	0.85
Pancreatic Guidewire Cannulation (%)	1 (3.1)	29 (10.7)	0.22
No Pancreatic Stent Placement (%)	32 (100)	252 (93.3)	0.23
Biliary Stenting (%)	2 (6.3)	94 (34.8)	***< 0.001***
Total Procedural Time ≥ 30 min (%)	15 (46.9)	148 (54.8)	0.46
Complete stone removal in the first session (%)	26 (81.3)	167 (61.9)	***0.03***
Post ERCP Pancreatitis (%)	5 (15.6)	10 (3.7)	***0.01***

^*^ Fisher’s exact test EST, sphincterotomy; EPLBD, endoscopic papillary large balloon dilation; EPBD, endoscopic papillary balloon dilation

### Diagnosis of asymptomatic CBDS

Of the 32 asymptomatic patients, 19 were diagnosed by CT, 11 by US imaging, and 2 by MR imaging. Twenty-three asymptomatic patients were accidentally diagnosed in imaging tests to evaluate other diseases (e.g., colorectal carcinoma and urinary calculus), and the remaining 9 patients were diagnosed in screening US.

### Diagnosis and grading of acute cholangitis

Of the 302 CBDS patients, 248 (82.1%) had acute cholangitis, and those with mild, moderate, and severe were 138 (55.6%), 101 (40.7%), and 9 (3.7%), respectively.

### ERCP procedures

The success rates of cannulation were 95.7% (289/302) in total, 87.5% in asymptomatic (28/32), and 96.6% in symptomatic (261/270). Deep cannulation time was more than 10 min in 115 patients (38.1%, including 13 cases for impossible deep cannulation). EPBD was performed in 98 patients (32.5%). Contrast injection into the pancreatic duct was performed in 167 (55.3%) and 30 patients (9.9%) who underwent cannulation using the pancreatic guidewire technique. Prophylactic pancreatic stent placement was performed in 18 patients. The procedure lasted for more than 30 min in 163 patients (54.0%). 

Biliary stenting was performed in 96 patients instead of complete stone removal, and the complete stone removal rate in the first session was 63.9%.

Of the 96 stenting patients, 77 received further ERCP sessions for stone removal during the first hospitalization (the complete stone removal rate was 93.4%), 15 were once discharged and re-hospitalized later for stone removal (the total complete stone removal rate was 98.6%), and 4 were biliary stenting without stone removal. Only one patient suffered from cholangitis caused by stent obstruction during this study period.

### ERCP-related complications

Complications occurred in 22 patients (22/316; 7.0%), including pancreatitis in 15 (4.7%), mild aspiration pneumonia in 1 (0.3%), and hemorrhage in 6 (1.9%). Of six hemorrhage cases, five were mild, and one was severe (needed angiographic emboli). Hemorrhage in these patients was due to the Mallory–Weiss syndrome (n = 3) and was after EST (n = 3). PEP was mild in 14 cases and severe in only 1 patient. All patients with ERCP-related complications were successfully managed. 

### Risk factors for PEP

The potential risk factors associated with PEP that were evaluated in this study are listed in Table 2. Univariate analysis identified deep cannulation time more than 10 min (p = 0.02), papillary treatment EPBD (p = 0.03), and asymptomatic CBDS (p = 0.01) as significant factors. Next, multivariate analysis revealed deep cannulation time more than 10 min (odds ratio (OR), 6.67; p < 0.001), papillary treatment EPBD (OR, 5.70; p < 0.001), and asymptomatic CBDS (OR, 5.49; p < 0.001) as independent risk factors for PEP (Table 3).

**Table 2.** Univariate Analyses to Detect Risk Factors for PEP.

Variable	With PEP (n = 15)	Without PEP (n = 287)	*p* value
Female Sex (%)	7 (46.7)	153 (53.3)	1^＊^
Age < 55 years (%)	2 (13.3)	34 (11.8)	0.70
Maximum Stone Diameter ≥ 10 mm (%)	2 (13.3)	70 (24.4)	0.53
Number of Stones ≥ 2 (%)	8 (53.3)	142 (49.5)	0.80
Diameter of CBD < 10 mm (%)	13 (86.7)	191 (66.6)	0.16
Serum Bilirubin < 1.5 mg/dL (%)	7 (46.7)	153 (53.3)	1
No Pre-Procedural Diclofenac (%)	13 (86.7)	252 (87.8)	1
Precut Sphincterotomy (%)	0	12 (4.2)	1
Deep Cannulation Time ≥ 10 min (%)	10 (66.7)	105 (36.6)	***0.02***
Papillary Treatment EPBD (%)	9 (60)	89 (31.0)	***0.03***
Contrast Injection into Pancreatic Duct (%)	10 (66.7)	157 (54.7)	0.43
Pancreatic Guidewire Cannulation (%)	3 (20)	27 (9.4)	0.18
No Pancreatic Stent Placement (%)	14 (93.3)	270 (97.1)	1
Biliary stenting (%)	5 (33.3)	91 (31.7)	1
Total Procedural Time ≥ 30 min (%)	8 (53.3)	155 (54.0)	1
Complete stone removal in the first session (%)	9 (60)	184 (64.1)	0.79
Asymptomatic CBDS (%)	5 (33.3)	27 (9.4)	***0.01***

^＊^Fisher’s exact test PEP, post-ERCP pancreatitis; OR, odds ratio; CBD, common bile duct; EST, sphincterotomy; EPLBD, endoscopic papillary large balloon dilation; EPBD, endoscopic papillary balloon dilation

**Table 3.** Multivariate Analyses to Detect Independent Risk Factors for PEP.

Variable	OR (95% CI)	*p* value
Deep Cannulation Time ≥ 10 min	6.67 (1.95–22.8)	***< 0.001***
Papillary Treatment EPBD	5.70 (1.75–18.6)	***< 0.001***
Asymptomatic CBDS	5.49 (1.58–19.1)	***< 0.001***

PEP, post-ERCP pancreatitis; OR, odds ratio; EPBD, endoscopic papillary balloon dilation; CBD, common bile duct

## Discussion

We showed that deep cannulation time more than 10 min, papillary treatment EPBD, and asymptomatic CBDS are significant independent risk factors for PEP. Risk factors for PEP can be categorized as patient-related and procedure-related. Thus, although asymptomatic CBDS is a patient-related factor, deep cannulation time and papillary treatment represent a procedure-related risk. 

The relationship among these independent risk factors and PEP may be explained as follows. Papillary edema is associated with difficult cannulation, and it leads to blockage of pancreatic juice flow and subsequent activation of trypsin and neutrophils, and finally PEP. The mechanism of EPBD-related PEP is still unclear. Damage to the pancreatic duct during papillary dilatation and papillary edema or spasm after dilatation is potentially associated with the induction of PEP ^(18)^. Generally, a smaller papillary orifice is related to difficulties in biliary cannulation ^(19)^. Compared to symptomatic CBDS, the papillary orifice might be smaller in asymptomatic CBDS because of low bile duct pressure secondary to the absence of cholestasis. However, we found no differences in deep cannulation time between asymptomatic and symptomatic CBDS patients, and the reason for this observation remains unknown. 

Endoscopic stone removal requires papillary treatment, and compared to EST, EPBD for biliary stone removal is associated with a greater risk of PEP ^(20)^. Our results show the same result, so EPBD may be avoided, especially in case of asymptomatic or if difficult for bile duct cannulation or if the damage by EPBD is localized to the papilla, and the placement of a prophylactic pancreatic stent could prevent EPBD-related PEP. 

In this cohort, a total of 96 patients underwent biliary stenting. Patients who underwent stent placement (without complete stone removal) might be at a lower risk of PEP than those who underwent complete stone removal. Single-stage endoscopic stone removal after a long cannulation time increased the incidence of PEP compared with the two-stage stone removal procedure ^(21)^. Biliary stent placement cannot decrease time for cannulation but can save the subsequent procedure and stone removal time. These may be the reasons of the lower risk of PEP in the biliary stent group. In this study, there is no significant difference in PEP rate between single-stage stone removal with long cannulation time and without long cannulation time.

Can high-risk patients of PEP in the asymptomatic group be selected preoperatively? Saito et al. reported that precut sphincterotomy, biliary balloon sphincter dilation, and trainee endoscopists were significant risk factors for PEP in 168 patients with asymptomatic CBD stones ^(22)^. We performed the same analysis in 32 asymptomatic cases, and serum bilirubin (p = 0.008) and papillary treatment EPBD (p = 0.05) were extracted in the univariate, but no significant factor was extracted in the multivariate.

In this study, 14 patients (all symptomatic) had no CBD stone based on ERCP (passing case). Fortunately, none of them had PEP, but it was a useless risk. Maruta et al. reported the efficacy of the endoscopic ultrasound (EUS)-first approach to avoid unnecessary ERCP ^(23)^. We will precede EUS for asymptomatic or high-risk patients to avoid unnecessary ERCP.

Many countries ^(2), (3), (4)^, including Japan ^(5)^, recommend endoscopic treatment of CBDS whenever it is detected and even when it is asymptomatic, but PEP incidence was 9.7%, and its mortality rate was 0.15% in a recent systematic review ^(24)^. The natural history of asymptomatic CBDS is not well known. A recent report in 77 asymptomatic CBDS patients using a wait-and-see approach reported the cumulative incidence of biliary complications to be 6.1% at 1 year, 11% at 3 years, and 17% at 5 years, with the disappearance of stones observed in 22 patients (19%) ^(25)^. Therefore, in asymptomatic CBDS, a wait-and-see approach may be an option. At least, explanation of high risk before treatment, adjustment of drug, and infusion volume assuming pancreatitis after treatment are necessary.

The limitations of this study include its retrospective design and the fact that it is a single-center study with a limited sample size. 

In conclusion, we report that longer deep cannulation time, EPBD, and asymptomatic CBDS are risk factors for PEP. A wait-and-see approach may be an option for the management of asymptomatic CBDS. EPBD may be avoided, especially in case of asymptomatic or if difficult for bile duct cannulation. Our research is exploratory, so prospective studies that evaluate the risk–benefit balance of early endoscopic removal of asymptomatic CBDS are required to validate these results.

## Article Information

### Conflicts of Interest

None

### Author Contributions

MK was the principal investigator, drafted the manuscript, and approved the final version of the manuscript. YT, HY, TY, TO, and KT contributed to the acquisition of data, reviewed the draft manuscript, and approved the final version of the manuscript. FA supervised the research project, reviewed the draft manuscript, and approved the final version of the manuscript.

### Approval by Institutional Review Board (IRB)

Approval code: 31-28

Name of institution: Kofu Municipal Hospital

Date of approval: March 30, 2020
